# The Structural Integrity of Plasmid-Encoded Pgp3 Is Essential for Induction of Hydrosalpinx by *Chlamydia muridarum*

**DOI:** 10.3389/fcimb.2019.00013

**Published:** 2019-02-05

**Authors:** Yumeng Huang, Yina Sun, Tai Qin, Yuanjun Liu

**Affiliations:** ^1^Department of Endocrinology and Metabolism, Tianjin Medical University General Hospital, Tianjin, China; ^2^Key Laboratory of Hormones and Development (Ministry of Health), Tianjin Key Laboratory of Metabolic Diseases, Tianjin Metabolic Diseases Hospital and Tianjin Institute of Endocrinology, Tianjin Medical University, Tianjin, China; ^3^Key Laboratory of Cancer Prevention and Therapy, Department of Pancreatic Cancer, National Clinical Research Center for Cancer, Tianjin Medical University Cancer Institute and Hospital, Tianjin, China; ^4^Department of Dermatovenereology, Tianjin Medical University General Hospital, Tianjin, China

**Keywords:** Pgp3, fragment deficiency, *Chlamydia muridarum*, hydrosalpinx, virulence factor

## Abstract

Pgp3 consists of globular N- and C-terminal domains connected by a triple-helical coiled-coil middle domain. We demonstrated previously that Pgp3 is required for induction of hydrosalpinx by *Chlamydia muridarum*. We constructed *C. muridarum* transformants harboring deletion of the Pgp3 N-terminus (pgp3Δn), C-terminus (pgp3Δc), or middle domain (pgp3Δm). C3H/HeJ and CBA/J mice infected with pgp3Δn or pgp3Δm failed to induce hydrosalpinx in oviduct tissue. However, the pgp3Δc transformant induced mild hydrosalpinx in 20% of C3H/HeJ mice (severity score 0.2 ± 0.6) and in 40% of CBA/J mice (severity score 0.8 ± 1.3). The attenuated pathogenicity of the transformants harboring Pgp3 domain deletions was correlated with impaired *in vitro* growth and significantly reduced infectivity in the mouse lower genital tract. Moreover, the oviduct tissue of C3H/HeJ and CBA/J mice infected with the Pgp3-domain-deficient transformants displayed less inflammatory cell infiltration. Thus, the structural integrity of plasmid-encoded Pgp3 is essential for induction of hydrosalpinx by *C. muridarum*.

## Introduction

*Chlamydia trachomatis* infection starts in the vagina and can ascend into the upper genital tract (UGT). It is usually asymptomatic and thus easily missed. However, *C*. *trachomatis* infection can lead to severe complications, such as ectopic pregnancy and infertility (Ault et al., 1998; Mishori et al., 2012; Hafner, 2015). The mechanisms by which *C. trachomatis* gives rise to oviduct pathology are unclear, and characterization of its virulence factors is hampered by the mild pathology induced in mice (Carmichael et al., 2013; Ramsey et al., 2014). *Chlamydia muridarum*, which can induce hydrosalpinx of the UGT in mice after intravaginal infection, is widely used in mechanistic studies of chlamydial pathogenesis (Lu et al., 2013; Campbell et al., 2014; Gupta et al., 2014; Lei et al., 2014; Sun et al., 2015).

Both *C. trachomatis* and *C. muridarum* contain a highly conserved, cryptic plasmid encoding eight open reading frames (ORFs), Pgp1–Pgp8. The native plasmid of both chlamydial species modulates virulence and infectivity in mice and in lower primates (O'connell et al., 2007; Lei et al., 2014; Sigar et al., 2014; Qu et al., 2015). Pgp1, Pgp2, Pgp6, and Pgp8 are essential for plasmid maintenance, as their stable transformants cannot be acquired if either of the ORFs is deleted from the plasmid shuttle; in contrast, Pgp3, Pgp4, Pgp5, or Pgp7 can be deleted from *C. trachomatis* serovar L2 and *C. muridarum*. Furthermore, Pgp4 controls the expression of plasmid- and some chromosome-encoded genes (Gong et al., 2013; Song et al., 2013). Pgp3 is involved in induction of oviduct pathology, and Pgp3-deficient *C. muridarum* does not induce hydrosalpinx in mice following intravaginal infection. The Pgp3-deficient *C. trachomatis* serovar L2 exhibits reduced infectivity after intravaginal inoculation (Ramsey et al., 2014). Pgp5 also contributes to *C. muridarum* pathogenesis in the UGT, albeit to a lesser degree than Pgp3 (Huang et al., 2015). Interestingly, Pgp5 deletion resulted in overexpression of several plasmid- and chromosome-encoded genes (Liu et al., 2014a).

We investigated the possible reasons to why Pgp3-deficient *C. muridarum* cannot induce hydrosalpinx in oviduct of mouse model. Pgp3 deficiency reduced its survival in the lower genital tract (LGT), impaired ascent to the UGT, and decreased invasion of oviduct tissue (Liu et al., 2014b). As the only plasmid-encoded protein secreted into the lumen of inclusion bodies and the cytosol, Pgp3 is an immunodominant chlamydial antigen (Wang et al., 2010; Budrys et al., 2012). The N-terminal domain of *C. trachomatis* Pgp3 contains a series of structural motifs commonly found in trimeric viral proteins, while the trimeric C-terminal domain is structurally similar to that of tumor necrosis factor (TNF) family cytokines (Galaleldeen et al., 2013). Because the *pgp3* sequence is highly conserved, the structure of *C. muridarum* Pgp3 shows nearly 84% similarity to that of *C. trachomatis* Pgp3.

Here we generated *C. muridarum* transformants harboring deletions in the Pgp3 N-terminal, middle, and C-terminal domains. We investigated the roles of these domains in the induction of hydrosalpinx, *in vitro* growth and infection of the mouse.

## Materials and Methods

### Chlamydia Organisms and Cell Lines

HeLa (human cervical epithelial carcinoma cells) cells used in this study were kindly provided by the Institute of Dermatology (PUMC, Nanjing, PRC). *Chlamydia muridarum* strains including the wild type *C. muridarum Nigg* strain (WT), the plasmid free (CMUT3), the intact plasmid transformant (Intact), the pgp3 deletion mutant (Δpgp3) [from Dr. Guangming Zhong's lab at the University of Texas Health Science Center at San Antonio, USA] were propagated, purified, aliquoted, and stored as described previously in the reference (Zhong et al., 2001). The new Pgp3 domain deletions were modified from the intact plasmid transformant as described below. For chlamydial infection, cells grown in 24-well plates with or without coverslips, 6-well plates or flasks containing DMEM (Gibco, New York, USA) with 10% fetal bovine serum (FBS, Institute of Hematology, CAMS &PUMC, Tianjin, China) at 37°C in an incubator supplied with 5% CO2 were inoculated with chlamydial organisms as described previously (Zhong et al., 2001).

### Generating *C. muridarum* Transformants of pgp3 Domain Deletion

For making pgp3 domain deleted mutants, primers listed in Table 1 were used to amplify DNA fragments lacking different pgp3 domain from the plasmid pGFP::CM by PCR using AccuPrime pfx SuperMix (Life technologies, Grand Island, NY). The desired PCR products were fused to produce the appropriate plasmids using the in-fusion HD cloning kit as described (Liu et al., 2014a). Plasmids were extracted from bacterial colonies with GFP and the extracted plasmids were partially digested by BamHI and XhoI. Plasmid with the desired fragments after digestion was fully sequenced and transformed into *E. coli* K12 ER2925 for amplification. The amplified plasmids designated as pGFP::CM pgp3Δn, pGFP::CM pgp3Δm or pGFP::CM pgp3Δc were used for transforming chlamydial organisms.

**Table 1:** The primers for construction of the Pgp3 domain deleted mutants.

	**Sense primer sequences**	**Antisense primer sequence**	**Tm(^**°**^C)**
Pgp3Δn	5′gcatctaatccaatatttaccatttgcccaact ttaatattgtcggcaa3′	5′atattaaagttgggcaaatggtaaatattggattagatgct gaaaaagcg3′	55
Pgp3Δm	5′ttgctagatgtgaataggcctgaagagttagctt gagcattgtttgttat 3′	5′atgctcaagctaactcttcaggcctattcacatcta gcaatgtaacaac 3′	55
Pgp3Δc	5′aattccaataaaattatttagttacattgaatttt cccagtgatttggaag3′	5′ctgggaaaattcaatgtaactaaataattttattggaat tttcttatcggt 3′	55

The plasmid of pGFP::CM pgp3Δn, GFP::CM pgp3Δm or pGFP::CM pgp3Δc was introduced into the plasmid-free *C. muridarum* strain CMUT3 in the form of a purified EB by following the protocol published previously (Liu et al., 2014a). The organisms of CMUT3-pGFP::CM pgp3Δn, CMUT3-pGFP::CM pgp3Δm or CMUT3-pGFP::CM pgp3Δc were plaque-purified as described previously (Zhong et al., 2001) for *in vitro* and *in vivo* experiments as described below.

### *In vitro* Characterization of *C. muridarum* Transformants

An immunofluorescence assay was used to detect Pgp3 and *C. muridarum* by a triple staining technique as described previously (Gong et al., 2013). Briefly, infected HeLa cells monolayer grown on coverslips were fixed with 4% paraformaldehyde for 30 min at room temperature, followed by permeabilization with 2% (wt/vol) saponin (Sigma) for an additional 60 min and then blocking. The cell samples were incubated with antibody and chemical staining. Hoechst (blue; Sigma) was used to mark DNA. Rabbit antibodies against *C. muridarum* plus secondary antibody conjugated with Cy2 (green; Abnova) was used to mark chlamydial inclusions. Mouse primary antibodies against Pgp3 protein in combination with a secondary antibody conjugated with Cy3 (red; Abnova) were used to mark the Pgp3 protein. Immunofluorescence images were acquired by using a confocal laser scanning microscope (Leica, Germany) and processed using Leica confocal software.

### Plaque Size Assay for *C. muridarum* Organisms

Plaque size assay was carried out for evaluating *in vitro* growth properties of *C. muridarum* organisms as described previously (Huang et al., 2015). Briefly, *Chlamydia muridarum* organisms were inoculated onto McCoy cells monolayer in 12-well plates and centrifuged at 1,200 rpm for 1 h at room temperature (RT). Then infected cells were cultured with overlay medium (1 × Dulbecco's modified Eagle's medium, 10% fetal bovine serum, 1 μg/ml cycloheximide, and a final concentration of 0.55% of agarose). The cells were allowed to incubate at 37°C in an atmosphere of 5% CO2 for 5 days before stained with 0.03% Neutral Red for 1 h at RT. After taking pictures, the diameters of plaques were measured with the custom MATLAB program plaque Detector, which can be freely accessed at http://www.mathworks.com/matlabcentral/fileexchange/48860-plaque-detector.

### Detection of the Genomic Copy Number After a Single Step Infection for *C. muridarum* Organisms

All *C. muridarum* organisms were inoculated onto HeLa monolayers in 6-well plates at an MOI of 0.8. The infected cells were harvested at the 24th h after infection. The cells before and after infection were lysed with 0.1% sodium dodecyl sulfate (SDS) and used as PCR templates for titrating the changes of genomic copy number using primers designed from16s rRNA gene. The sequences of primers were listed as follows: P1 5′ cgcctgaggagtacactcgc 3′, P2 5′ ccaacacctcacggcacgag 3′. The experiment was repeated three times with duplicate in each.

### Mouse Infection and Live Organism Recovery From Vaginal Swabs

The wild type *C. muridarum* Nigg strain or plasmid-free *C. muridarum* CMUT3 with or without transformation with the full plasmid or plasmids with pgp3 different domain deletion was used to infect female C3H/HeJ mice or CBA/J intravaginally with 2 × 10^5^ inclusion-forming units (IFUs). To increase mouse susceptibility to the infections, each mouse was injected with 2.5 mg medroxyprogesterone (Depo-Provera; Pharmacia Upjohn, Kalamazoo, MI) subcutaneously 5 days prior to the infection.

To monitor live organism shedding from lower genital tract, vaginal swabs were taken on different days after infection. To quantitate live chlamydial organisms, each swab was dissolved in 500 μl of ice-cold SPG and vortexed with five glass beads, and the chlamydial organisms released into the supernatants were titrated on HeLa cell monolayers in duplicate as described previously (Liu et al., 2014b). The total number of IFUs per swab was calculated based on the number of IFUs per view, the number of views per coverslip, dilution folds, inoculation doses and total sample volumes. The calculated total number of IFUs/swab was converted into log_10_, and the log_10_ IFUs were used to calculate the mean and standard deviation at each time point. These animal experiments were carried out according to the principles of the Guide for the Care and Use of Laboratory Animals of the National Institutes of Health. The protocol was approved by the Ethics Committee of Tianjin Medical University General Hospital.

### Evaluating Mouse Genital Tract Tissue Pathologies and Histological Scoring

On the 60th day after infection, mice were sacrificed to harvest their entire genital tracts including the vagina, the uterus, the oviduct, and the ovary for evaluating tissue pathology. Before removing the genital tract tissues from the mice, an *in situ* gross examination was performed for evidence of oviduct hydrosalpinx or any other related abnormalities. The genital tract tissues were isolated entirely and laid on a blue background to acquire images. The oviduct hydrosalpinx were visually scored based on their dilation sizes using a scoring system as described previously (Chen et al., 2014). Mice with hydrosalpinx on either side of the oviducts were determined to be hydrosalpinx positive and the severity of hydrosalpinx was scored based on the following criteria: No oviduct dilation found with a stereoscope inspection is defined as no hydrosalpinx and a score of zero (0); Hydrosalpinx is visible only under stereoscope but not naked eyes (1); Hydrosalpinx is visible with naked eye but the size is smaller than the ovary (2); The size of hydrosalpinx is equal to the ovary (3) or larger than the ovary (4). Scores from both sides of the oviducts from the same mouse were combined as the total gross pathology score for that mouse. Both the incidence and severity scores of oviduct hydrosalpinx were statistically analyzed between mice infected with different *C. muridarum* organisms. The researchers who scored the pathology were blinded to the experimental groups.

For the observation of histological pathology, the isolated mouse genital tract tissues were fixed in 10% neutral formalin and embedded in paraffin and serially sectioned longitudinally. The sections were stained with hematoxylin and eosin (H&E) as described before (Liu et al., 2014b). The H&E stained sections were observed under microscope for severity of inflammation and pathologies based on the modified schemes established previously (Murthy et al., 2011). Scoring for inflammatory cell infiltration was as follows: 0, no significant infiltration; 1, infiltration at one single focus; 2, infiltration at two to four foci; 3, infiltration at more than four foci; 4, confluent infiltration. For observing oviduct dilation and inflammatory infiltration, image from each mouse was taken under a 40X and 100X objective lens.

### Statistics Analyses

Quantitative data including the diameters of plaque sizes was analyzed using Student's *t*-test while the overall IFU shedding in form of Log10 was analyzed using Kruskal-Wallis test. All semi-quantitative data including the pathology scores was analyzed using Mann–Whitney *U* rank-sum test. The qualitative data including incidence rates was analyzed using Fisher's exact test.

## Results

### Characterization of *C. muridarum* pgp3 Domain Deletion Mutants

**Figure 1:**
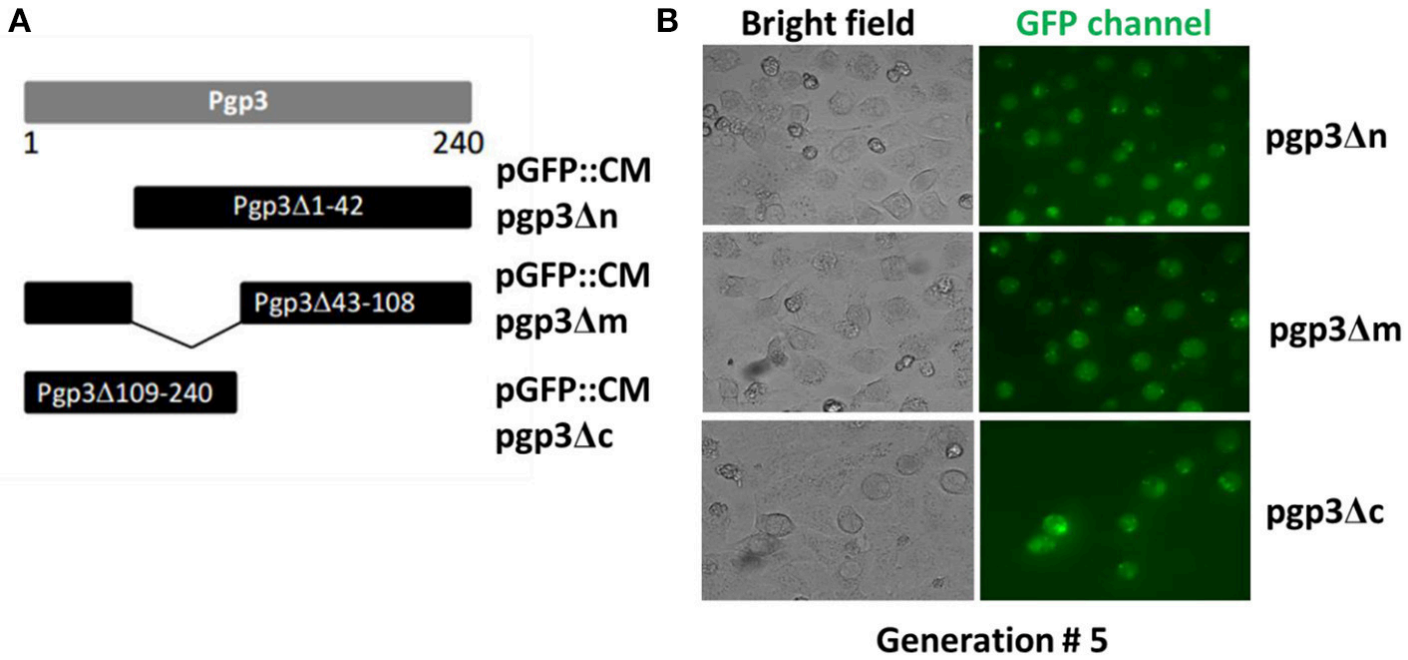
Construction of Pgp3 domain deletion mutants. **(A)** The Pgp3 N-terminal (amino acids [aa] 1–42), middle (aa 43–108), or C-terminal (aa 109–240) domain was deleted from the full length plasmid GFP::CM to generate pGFP::CM pgp3Δn, pGFP::CM pgp3Δm, or pGFP::CM pgp3Δc, respectively. **(B)** The plasmids harboring the Pgp3 domain deletions were transformed into a plasmid-free clone CMUT3 in the form of elementary bodies (EBs). The culture was incubated for 12 h without ampicillin and then with ampicillin for another 20 h. GFP-positive inclusion body pick-up selection and passage were repeated for 5 generations. The GFP-positive organisms were enriched. So the stable transformants pgp3Δn, pgp3Δm, and pgp3Δc were produced successfully.

We generated mutants harboring deletion constructs of either the *pgp3* N-terminus, middle domain, or C- terminus by modifying pGFP::CM, resulting in pGFP::CM pgp3Δn, pGFP::CM pgp3Δm, or pGFP::CM pgp3Δc, respectively (Figure 1A). These plasmids were transformed into the *C. muridarum* plasmid-free clone CMUT3, and the resulting strains were cultured with L929 cell monolayers for 12 h without ampicillin and subsequently with ampicillin for 20 h. After four to five passages, GFP-positive inclusions were enriched, and a single clone of each transformant was selected by plaque assay and purified. The CMUT3 transformants obtained were named CMUT3-pGFP::CM pgp3Δn, CMUT3-pGFP::CM pgp3Δm, and CMUT3-pGFP::CM pgp3Δc (Figure 1B), referred to hereafter as pgp3Δn, pgp3Δm, and pgp3Δc, respectively.

**Figure 2:**
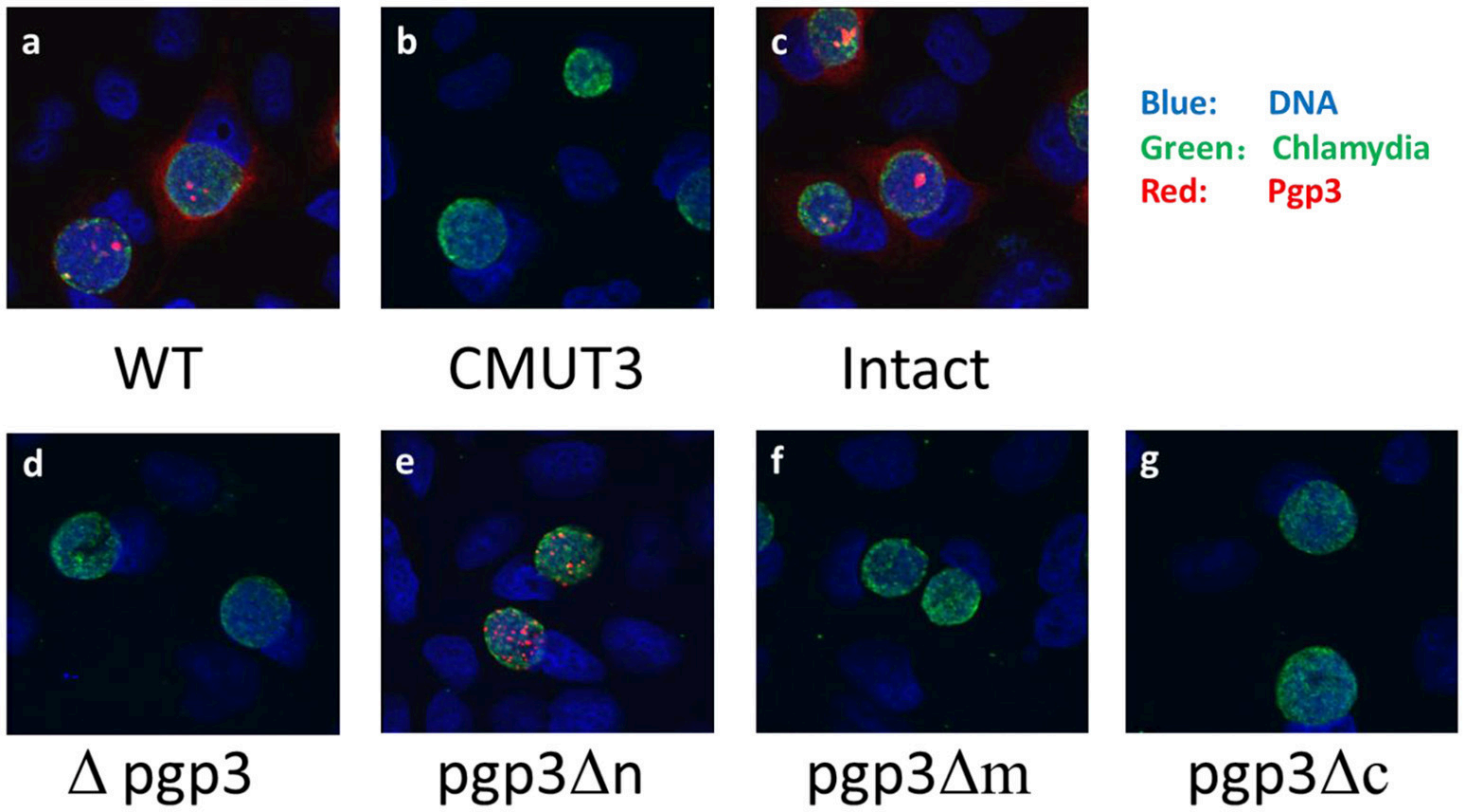
Effect of Pgp3 domain deletion on polyclonal antibody recognition of Pgp3. The wild-type *C. muridarum* **(a)**, plasmid-free clone CMUT3 **(b)**, intact pGFP::CM **(c)**, and pgp3 full-length deletion mutant (Δpgp3, **d**), three domain deletion mutants **(e–g)** were used to infect HeLa cells. The cells were subjected to triple immunofluorescence staining for pgp3 (red), chlamydia (green), and DNA (blue). Only pgp3Δn was recognized by the anti-Pgp3 polyclonal antibody. pgp3Δn was localized to the inclusion body, whereas intact Pgp3 was detected throughout the cytosol.

We assessed Pgp3 expression in the three stable transformants. *C. muridarum* Nigg (wild type [WT]), CMUT3, and CMUT3 transformed with pGFP::CM (CMUT3-pGFP::CM, intact) or with deletion of full-length *pgp3* (CMUT3-pGFP::CMΔpgp3 [Δpgp3]) were used as the controls. The plasmid-free CMUT3, Δpgp3, pgp3Δm, and pgp3Δc lacked the corresponding signals of Pgp3 (Figure 2, Figure S1A in the Supplemental Material). The failure to detect mutated Pgp3 in pgp3Δm and pgp3Δc organisms may be due to the absence of their epitopes, for the mutated Pgp3 expressions were detectable at transcriptional level (Figure S2). Besides, the His-tagged mutated Pgp3 expressions were verified in *E. coli* expression system (data not shown). The Pgp3 protein in pgp3Δn was detected only in chlamydial inclusion bodies, but not in host cell cytoplasm (Figure 2, Figure S1). We further performed a Western blot of native gel to compare the protein levels of Pgp3 in the three Pgp3 mutants, using intact organisms as positive control and Δpgp3 as a negative control. The result was consistent with that from immunofluorescence staining (Figure S1). Therefore, Pgp3 with deletion of its N-terminus was not secreted into the host cell cytoplasm, whereas the middle and C-terminal domains of Pgp3 are essential for polyclonal antibody recognition.

### Pgp3-Domain-Deficient *C. muridarum* Did Not Induce Severe Hydrosalpinx

**Figure 3:**
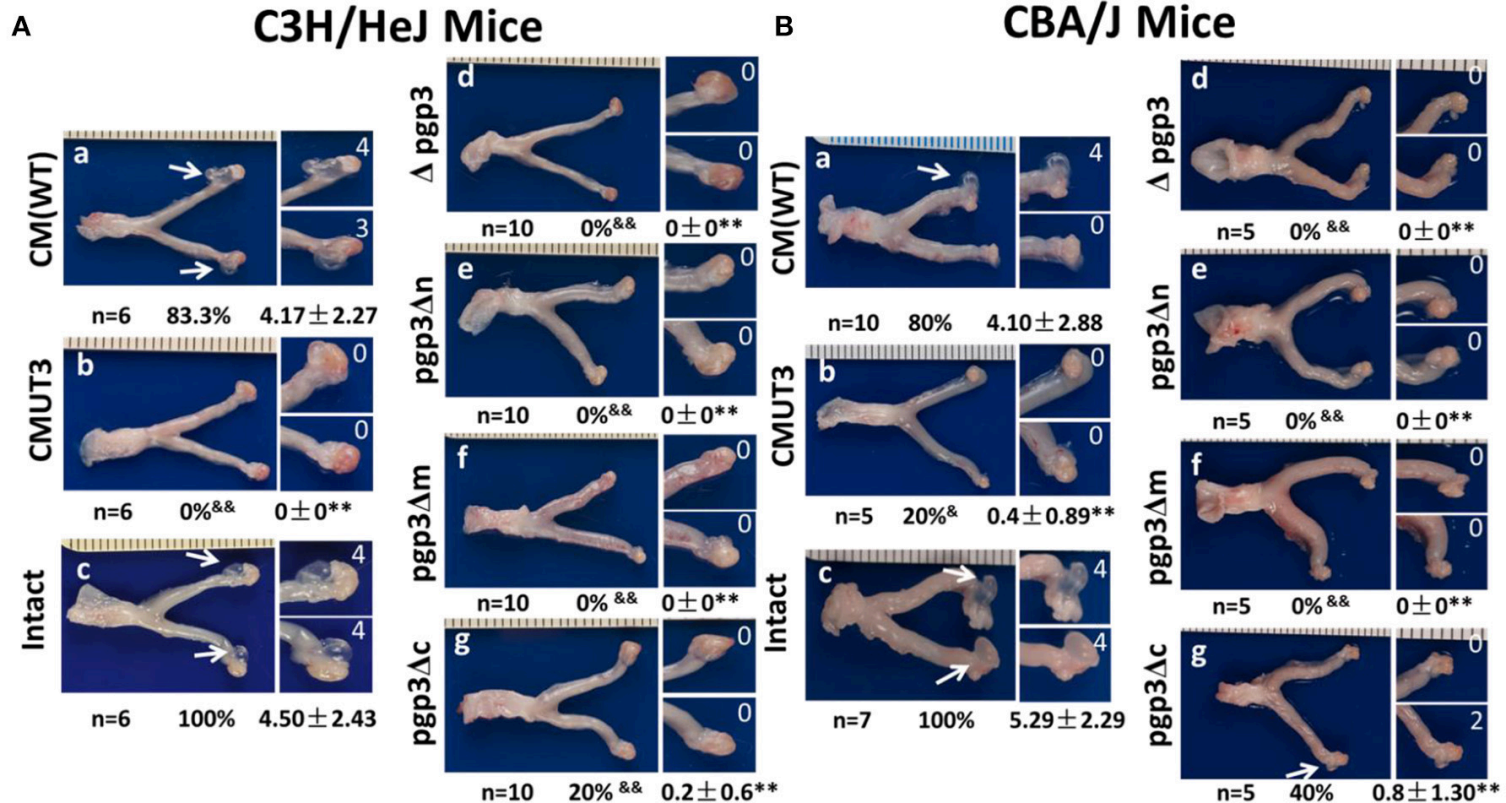
Effect of Pgp3 domain deletion on chlamydial pathogenesis. Wild-type *C. muridarum* (WT, a), plasmid-free (CMUT3, b) or CMUT3 transformants (pGFP::CM [intact], c; Δpgp3, d; pgp3Δn, e; pgp3Δm, f; pgp3Δc, g) were used to intravaginally infect C3H/HeJ **(A)** and CBA/J mice **(B)**. Sixty days after inoculation, the genital tracts were harvested, and the gross pathology in the UGT was evaluated. Left, representative image of the genital tract; right, representative image of the oviduct/ovary. The total number of mice (n) and the incidence (%) and severity (± SD) of hydrosalpinx are shown. pgp3Δn, pgp3Δm, and pgp3Δc did not induce severe hydrosalpinx. ^&^*P* < 0.05, ^&&^*P* < 0.01; Fisher's exact test. ^**^P < 0.01; Mann–Whitney *U* rank-sum test.

We evaluated the effect of Pgp3 domain deficiency on induction of hydrosalpinx by *C. muridarum*. C3H/HeJ mice were infected with WT, CMUT3, plasmid-competent (intact), Δpgp3, pgp3Δn, pgp3Δm, or pgp3Δc *C. muridarum* intravaginally. Sixty days after infection, the mice were euthanized and their genital tracts were harvested for assessment of oviduct pathology (Figure 3A). CMUT3 and Δpgp3 did not induce hydrosalpinx; in contrast, 80% of the C3H/HeJ mice infected with WT or intact *C. muridarum* developed severe hydrosalpinx (severity score 4.17 ± 2.27), which is consistent with previous reports (Lei et al., 2014; Liu et al., 2014b). However, pgp3Δn and pgp3Δm failed to induce this pathology in the mouse oviduct, and pgp3Δc induced hydrosalpinx in only 20% of C3H/HeJ mice (severity score 0.2 ± 0.6). Therefore, we then infected CBA/J mice, which are more susceptible to *C. muridarum* infection (Chen et al., 2014), with the above-mentioned *C. muridarum* strains. CMUT3 induced hydrosalpinx in 20% of the mice. Δpgp3, pgp3Δn, and pgp3Δm did not induce hydrosalpinx in CBA/J mice, whereas 40% of the mice infected with pgp3Δc showed mild pathology in the oviduct (severity score 0.8 ± 1.3). Therefore, the Pgp3 C-terminus is less important for *C. muridarum* pathogenicity than are the N-terminal and middle domains. However, deletion of any of the three Pgp3 domains significantly reduced the pathogenicity of *C. muridarum* in the mouse genital tract.

### Pgp3-Domain-Deficient *C. muridarum* Exhibited Reduced *in vitro* Growth and Survival in the Mouse LGT

**Figure 4:**
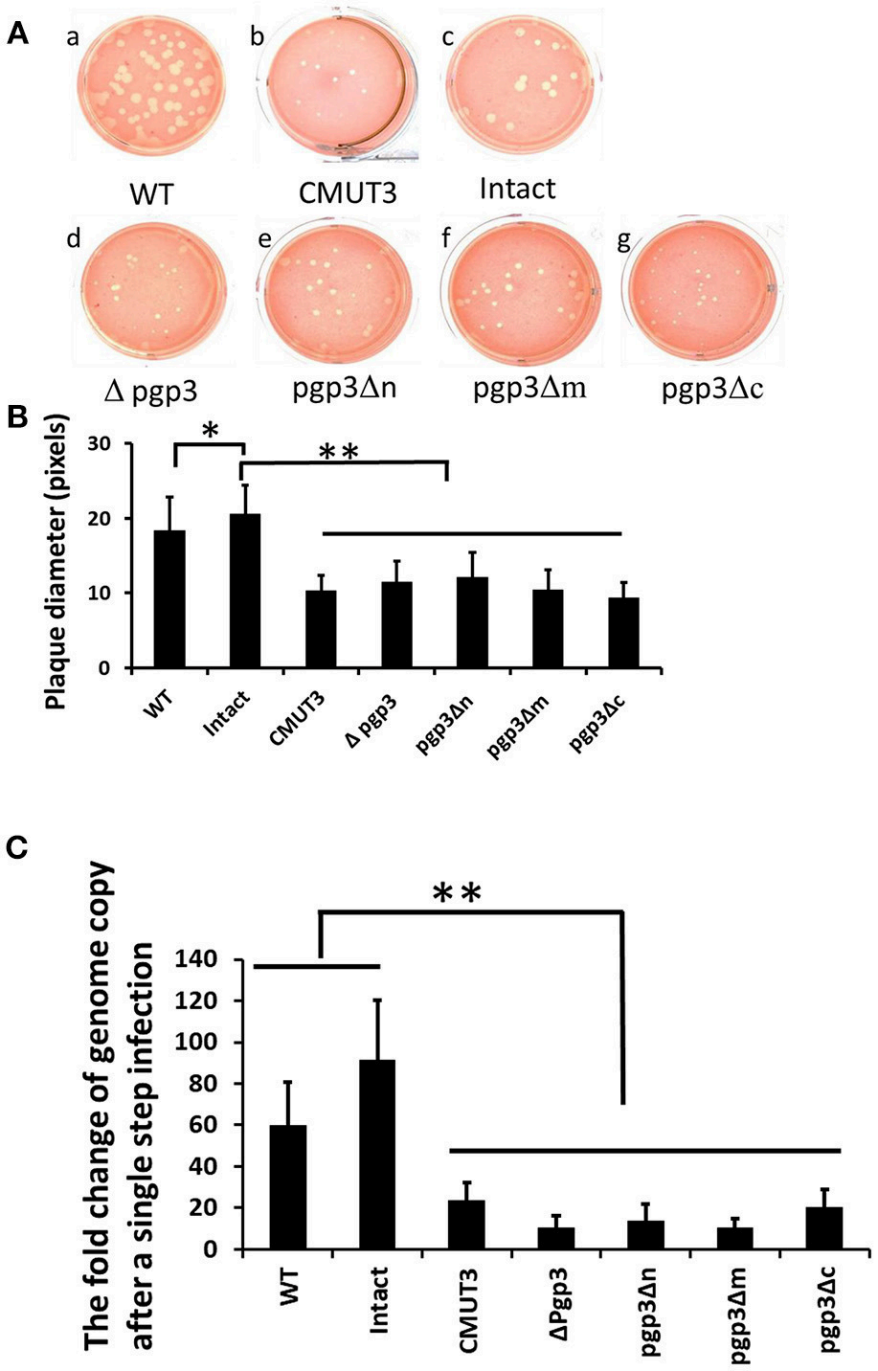
Effect of Pgp3 domain deletion on chlamydial growth *in vitro*. **(A)** Wild-type *C. muridarum* (WT, a); plasmid-free *C. muridarum* (CMUT3, b); CMUT3 transformed with pGFP::CM (intact, c) or pGFP::CM with pgp3 full-length deletion (Δpgp3, d), pgp3 N-terminus deletion (pgp3Δn, e), pgp3 middle domain deletion (pgp3Δm, f), or pgp3 C-terminus deletion (pgp3Δc, g) was stained with neutral red after inoculation onto McCoy cell monolayers in 12-well plates for 5 days. **(B)** Plaque sizes were measured in pixels using PlaqueDetector. Plasmid-free CMUT3, Δpgp3, pgp3Δn, pgp3Δm, and pgp3Δc produced significantly smaller plaques (^*^P < 0.05, ^**^P < 0.01; Student's *t*-test). **(C)** qPCR was used to measure the changes of genomic copy number of these strains after a single step infection. The growth rates of pgp3-domain-deficient transformants and plasmid free CMUT3 were significantly lower than those of WT or intact *C. muridarum* (^**^P < 0.01; Student's *t*-test).

Since the chlamydial infectivity seems to be correlated with its ability to induce oviduct pathology, we evaluated the sizes of plaques formed by the three transformants, which can reflect chlamydial infectivity, growth rate, and exit from the cell *in vitro*. The plaques formed by CMUT3 were significantly smaller than those formed by WT or intact *C. muridarum* (Figures 4A,B). The plaques formed by each of the three Pgp3-domain-deficient transformants were smaller than those formed by WT or intact *C. muridarum* (Figure 4). Thus, deletion of any of the three Pgp3 domains reduced the *in vitro* infectivity, growth rate, and exit from the cell of *C. muridarum*. Additionally, qPCR was used to measure the change of genomic copy after a single step infection. It displayed that the growth rates of Pgp3-domain-deficient transformants were much lower than those of WT or intact *C. muridarum* (Figure 4C).

**Figure 5:**
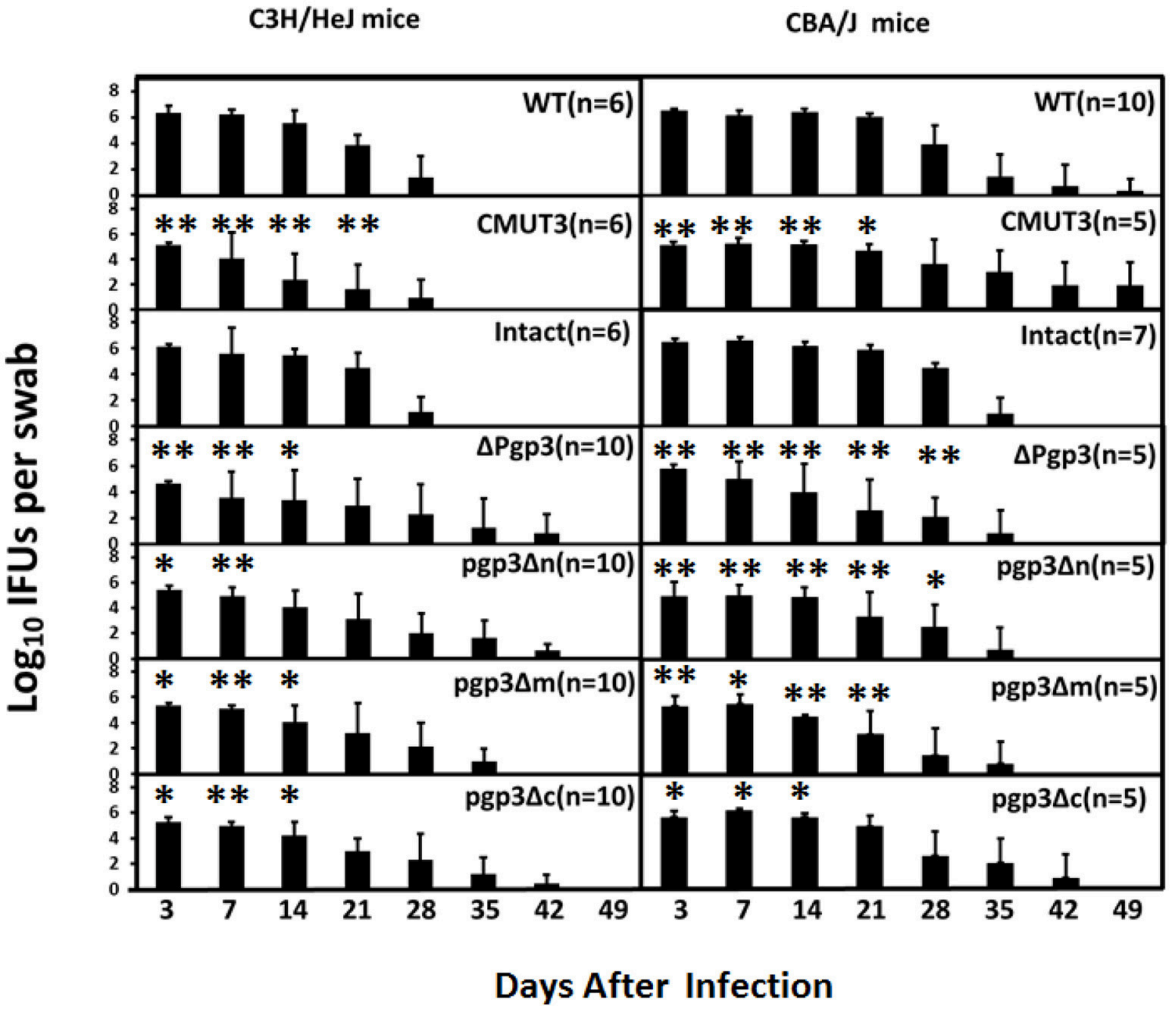
Effect of Pgp3 domain deletion on shedding from the LGT. C3H/Hej and CBA/J mice were intravaginally infected with *C. muridarum*. On the indicated days postinfection, vaginal swabs were harvested, and live organisms were enumerated by infecting HeLa cell monolayers; the results are presented in log10 IFUs. Mice infected with Δpgp3, pgp3Δn, pgp3Δm, pgp3Δc, or plasmid-free CMUT3 shed significantly smaller numbers of live organisms compared with mice infected with intact organisms. ^*^P < 0.05, ^**^P < 0.01; Kruskal–Wallis test.

In addition, live organisms shedding of *C. muridarum* from the LGT of infected mice was evaluated (Figure 5) as an indirect measure of survival *in vivo*. As expected, C3H/HeJ mice infected with CMUT3 *C. muridarum* showed significantly reduced shedding from the LGT at days 3, 7, 14, and 21 compared with mice infected with intact *C. muridarum*. The Δpgp3 *C. muridarum* resulted in significantly reduced shedding from the LGT at 3, 7, and 14 days postinfection. Importantly, pgp3Δm and pgp3Δc exhibited reduced *C. muridarum* shedding from the LGT at 3, 7, and 14 days postinfection, whereas pgp3Δn showed reduced shedding at 3 and 7 days post-infection.

The duration of vaginal shedding was slightly longer in CBA/J than in C3H/HeJ mice, possibly because CBA/J mice are more susceptible to *C. muridarum* infection. Shedding of live organisms by mice infected with CMUT3 or Δpgp3 *C. muridarum* was decreased at 3, 7, 14, and 21 days postinfection. Moreover, CBA/J mice infected with the three Pgp3-domain-deficient transformants showed significantly reduced shedding of live organisms. Shedding of pgp3Δn was reduced at 3, 7, 14, 21, and 28 days postinfection, and shedding of pgp3Δm and pgp3Δc was reduced at 3, 7 and 14 days postinfection. Therefore, each of the three Pgp3 domains is required for infection of the mouse LGT by *C. muridarum*.

### Pgp3-Domain-Deficient *C. muridarum* Exhibited Reduced Inflammation in the Mouse Oviduct

**Figure 6:**
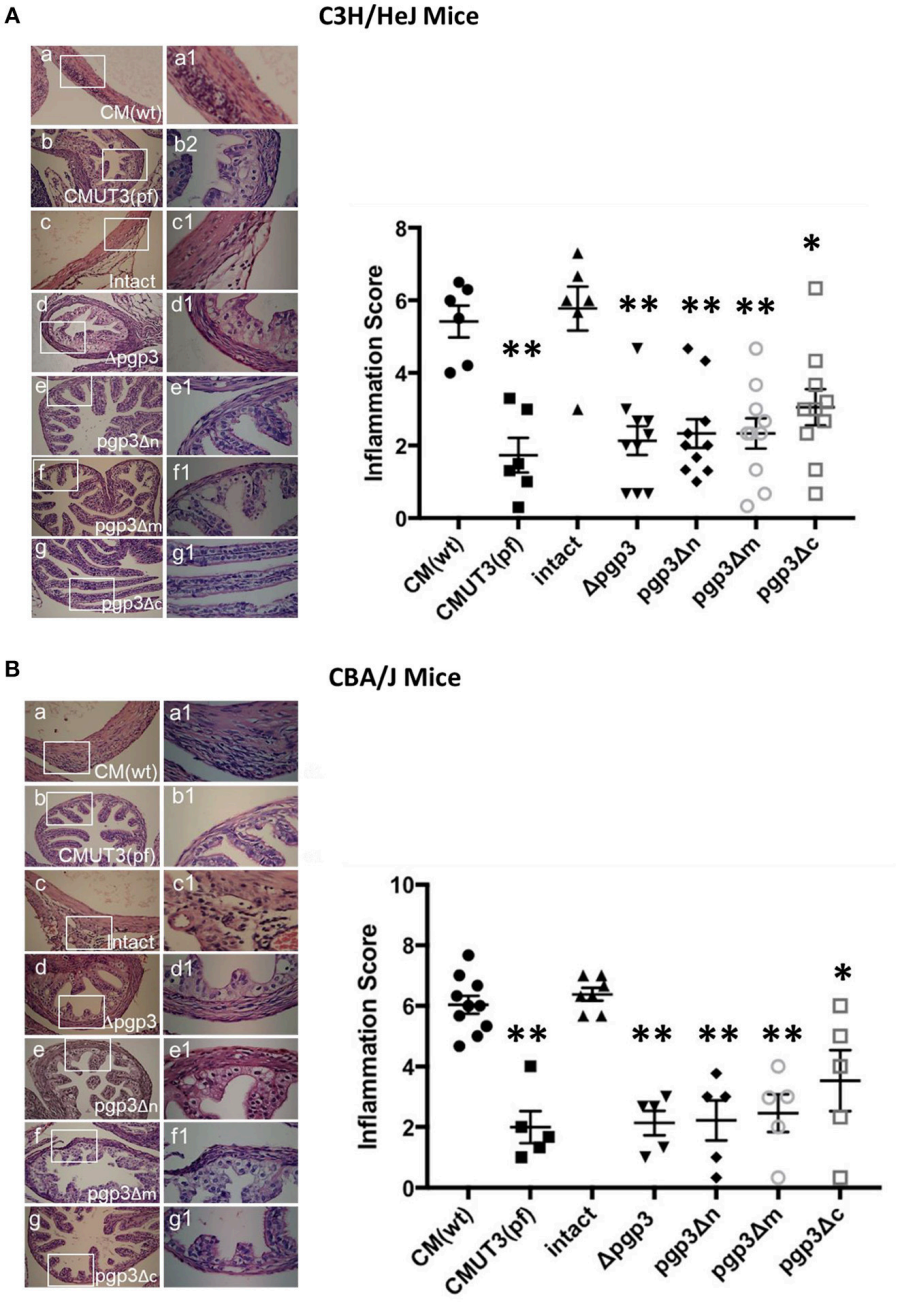
Effect of Pgp3 domain deletion on chlamydia-inducing histopathology. Ovary/oviduct tissues from C3H/HeJ **(A)** or CBA/J mice **(B)** were harvested and subjected to hematoxylin and eosin staining to evaluate inflammatory cell infiltration and oviduct dilation. (a–g) Representative image from each group at 40 × magnification; (a1–g1) enlargements (100 × magnification) of the areas demarcated by white rectangles. Inflammatory cell infiltration was semi-quantified (right) using intact organisms as the control. WT and intact *C. muridarum*, but not the pgp3-domain-deficient mutants, induced severe oviduct dilation and inflammatory cell infiltration. ^*^P < 0.05, ^**^P < 0.01; Mann–Whitney *U* rank-sum test.

The chlamydial infectivity and host inflammatory infiltrations in oviduct are two factors contributing to oviduct hydrosalpinx. On this basis, we supposed that the three Pgp3-domain-deficient transformants had impaired infectivity in mouse genital tract. However, the host immune response stimulated by chlamydia in oviduct still remains unknown. To settle this issue, we assessed inflammatory cell infiltration in the oviduct tissue of C3H/HeJ and CBA/J mice at 60 days post-intravaginal infection, as this is essential for hydrosalpinx induction by *C. muridarum* (Chen et al., 2014; Lei et al., 2014; Liu et al., 2014b; Huang et al., 2015). As expected, intact and WT *C. muridarum* induced markedly greater inflammatory cell infiltration in oviduct tissue compared with CMUT3 (Figure 6), which is consistent with our previous report. Interestingly, the transformants deficient in either of the three Pgp3 domains or full-length Pgp3 induced a lower level of inflammatory cell infiltration in the oviduct. In contrast, pgp3Δc induced slightly greater inflammatory cell infiltration (severity score 3.05 ± 1.49 for C3H/HeJ and 3.53 ± 2.24 for CBA/J mice) compared with CMUT3. These results were consistent with the gross pathology, indicating that pgp3Δc-induced hydrosalpinx may be due, in part, to relatively robust inflammatory cell infiltration in oviduct tissue.

WT and intact *C. muridarum* induced considerably greater oviduct dilation compared with CMUT3, Δpgp3, pgp3Δn, pgp3Δm, or pgp3Δc *C. muridarum*. These findings are consistent with the gross pathology (data not shown).

## Discussion

Pgp3 is important for the induction of hydrosalpinx by *C. muridarum* in the oviduct. Here, we report that all three Pgp3 domains are essential for its pathogenicity. We first investigated the effect of Pgp3 domain deletion on Pgp3 production and secretion. Only *C. muridarum* deficient in the Pgp3 N-terminus was recognized by an anti-Pgp3 polyclonal antibody (Figure 2 and Figure S1). Interestingly, Pgp3 lacking the N-terminus was not secreted into the host cell cytosol. The western blotting under non-reducing condition showed high molecular weight complexes in the pgp3Δn lane (Figure S1B). We suppose the complexes might have been formed by Pgp3Δn polymerization, for they did not present under reducing condition (data not shown). The polymerization may reduce Pgp3Δn secretion into host cell cytosol, which needs further investigation for confirmation. Since our anti-Pgp3 polyclone antibody failed to recognize Pgp3 lacking middle or C-domain, the mutated Pgp3 expressions were confirmed by RT-PCR assay (Figure S2). Therefore, the Pgp3 N-terminus is critical for secretion and the middle and C-terminal domains for antibody recognition. Deficiency in any of the three Pgp3 domains significantly reduced the incidence and severity of hydrosalpinx (Figures 3, 6). Second, either of Pgp3 domains deficiency affected *in vitro* growth and survival in the LGT (Figure 5). Third, after ascending into the oviduct, Pgp3-domain-deficient *C. muridarum* resulted in lower levels of inflammatory cell infiltration (Figure 6). Although the cause of the decreased oviduct inflammation is unclear, our data showed that the structural integrity of Pgp3 is essential for induction of hydrosalpinx by *C. muridarum*.

Pgp3 is the most important plasmid-encoded virulence factor for *C. muridarum* to induce hydrosalpinx in mice as it promotes chlamydial establishment in the LGT and ascent to the UGT (Liu et al., 2014b). However, the Pgp3 domain(s) associated with virulence is unknown. Here, we demonstrated that the structural integrity of the Pgp3 domains is necessary for the virulence of *C. muridarum*. For the induction of oviduct hydrosalpinx, the transformants with Pgp3 N-terminus or middle domain deletion phenocopy pgp3 full length deletion organisms. Interestingly, organisms with Pgp3 C-terminus deletion induced mild pathology in the oviduct tissue of 20% of C3H/HeJ and 40% of CBA/J mice (Figure 3). Therefore, the C-terminal domain of Pgp3 is less important than the other two domains for the pathogenesis of *C. muridarum*. The structure of the C-terminus resembles that of TNF proteins, suggesting that it interacts with host inflammatory factors. Although *C. muridarum* infectivity in the mouse LGT is correlated with induction of hydrosalpinx in the oviduct, deletion of the Pgp3 C-terminus did not show enhanced infectivity in the mouse LGT than the other two Pgp3 domains deleted transformants.

As inflammation is also another critical element for chlamydial induction of hydrosalpinx, we assessed inflammatory cell infiltration in oviduct tissue; deletion of any of the three Pgp3 domains suppressed *C. muridarum*–induced inflammatory cell infiltration. During *C. trachomatis* infection, Pgp3 is presented to the immune system as a trimer (Chen et al., 2010); therefore, Pgp3-domain-deficient monomers might not be able to form trimers and fail to stimulate robust immune responses. However, this hypothesis needs further confirmation. Moreover, *C. trachomatis* Pgp3 neutralizes the anti-chlamydial activity of LL-37, a host antimicrobial peptide secreted by genital tract epithelial cells and infiltrating neutrophils (Hou et al., 2015). It is possible that the mutant Pgp3 proteins deficient in one of the three Pgp3 domains have reduced anti-chlamydial activity, which would explain the reduced infectivity in the mouse LGT. Comparing inflammation score in each group, although CMUT3, Δpgp3, pgp3Δn, pgp3Δm, and pgp3Δc induced low level of inflammatory cell infiltration, pgp3Δc showed slightly more severe inflammatory cell infiltration than others (Figure 6). Therefore, the ability of pgp3Δc to induce mild hydrosalpinx may be due to enhanced inflammatory cell infiltration, but not promotion of infectivity. Further investigation is needed to identify the immune factor(s) that act independently of Pgp3 or its domains.

## Author Contributions

YH, YS, and YL performed the experiments. TQ analyzed the data. YH wrote the paper.

### Conflict of Interest Statement

The authors declare that the research was conducted in the absence of any commercial or financial relationships that could be construed as a potential conflict of interest.

## References

[B1] Ault K. A. Statland B. D. King M. M. Dozier D. I. Joachims M. L. Gunter J. (1998). Antibodies to the chlamydial 60 kilodalton heat shock protein in women with tubal factor infertility. Infect. Dis. Obstet. Gynecol. 6, 163–167. 10.1002/(SICI)1098-0997(1998)6:4<163::AID-IDOG5>3.0.CO;2-6 9812248 PMC1784801

[B2] Budrys N. M. Gong S. Rodgers A. K. Wang J. Louden C. Shain R.. (2012). Chlamydia trachomatis antigens recognized in women with tubal factor infertility, normal fertility, and acute infection. Obstet. Gynecol. 119, 1009–1016. 10.1097/AOG.0b013e3182519326 22525912 PMC4608258

[B3] Campbell J. Huang Y. Liu Y. Schenken R. Arulanandam B. Zhong G. (2014). Bioluminescence imaging of *Chlamydia muridarum* ascending infection in mice. PLoS ONE 9:e101634. 10.1371/journal.pone.0101634 24983626 PMC4077820

[B4] Carmichael J. R. Tifrea D. Pal S. De La Maza L. M. (2013). Differences in infectivity and induction of infertility: a comparative study of *Chlamydia trachomatis* strains in the murine model. Microbes Infect. 15, 219–229. 10.1016/j.micinf.2012.12.001 23287699 PMC3602122

[B5] Chen D. Lei L. Lu C. Galaleldeen A. Hart P. J. Zhong G. (2010). Characterization of Pgp3, a *Chlamydia trachomatis* plasmid-encoded immunodominant antigen. J. Bacteriol. 192, 6017–6024. 10.1128/JB.00847-10 20851898 PMC2976438

[B6] Chen J. Zhang H. Zhou Z. Yang Z. Ding Y. Zhou Z.. (2014). Chlamydial induction of hydrosalpinx in 11 strains of mice reveals multiple host mechanisms for preventing upper genital tract pathology. PLoS ONE 9:e95076. 10.1371/journal.pone.0095076 24736397 PMC3988139

[B7] Galaleldeen A. Taylor A. B. Chen D. Schuermann J. P. Holloway S. P. Hou S.. (2013). Structure of the *Chlamydia trachomatis* immunodominant antigen Pgp3. J. Biol. Chem. 288, 22068–22079. 10.1074/jbc.M113.475012 23703617 PMC3724661

[B8] Gong S. Yang Z. Lei L. Shen L. Zhong G. (2013). Characterization of *Chlamydia trachomatis* plasmid-encoded open reading frames. J. Bacteriol. 195, 3819–3826. 10.1128/JB.00511-13 23794619 PMC3754608

[B9] Gupta R. Wali S. Yu J. J. Chambers J. P. Zhong G. Murthy A. K. (2014). *In vivo* whole animal body imaging reveals colonization of *Chlamydia muridarum* to the lower genital tract at early stages of infection. Mol. Imaging Biol. 16, 635–641. 10.1007/s11307-014-0732-5 24723309

[B10] Hafner L. M. (2015). Pathogenesis of fallopian tube damage caused by *Chlamydia trachomatis* infections. Contraception 92, 108–115. 10.1016/j.contraception.2015.01.004 25592078

[B11] Hou S. Dong X. Yang Z. Li Z. Liu Q. Zhong G. (2015). Chlamydial plasmid-encoded virulence factor Pgp3 neutralizes the antichlamydial activity of human cathelicidin LL-37. Infect. Immun. 83, 4701–4709. 10.1128/IAI.00746-15 26416907 PMC4645396

[B12] Huang Y. Zhang Q. Yang Z. Conrad T. Liu Y. Zhong G. (2015). Plasmid-encoded Pgp5 is a significant contributor to *Chlamydia muridarum* induction of hydrosalpinx. PLoS ONE 10:e0124840. 10.1371/journal.pone.0124840 25915629 PMC4411118

[B13] Lei L. Chen J. Hou S. Ding Y. Yang Z. Zeng H.. (2014). Reduced live organism recovery and lack of hydrosalpinx in mice infected with plasmid-free *Chlamydia muridarum*. Infect. Immun. 82, 983–992. 10.1128/IAI.01543-13 24343644 PMC3958014

[B14] Liu Y. Chen C. Gong S. Hou S. Qi M. Liu Q.. (2014a). Transformation of *Chlamydia muridarum* reveals a role for Pgp5 in suppression of plasmid-dependent gene expression. J. Bacteriol. 196, 989–998. 10.1128/JB.01161-13 24363344 PMC3957687

[B15] Liu Y. Huang Y. Yang Z. Sun Y. Gong S. Hou S.. (2014b). Plasmid-encoded Pgp3 is a major virulence factor for *Chlamydia muridarum* to induce hydrosalpinx in mice. Infect. Immun. 82, 5327–5335. 10.1128/IAI.02576-14 25287930 PMC4249284

[B16] Lu C. Peng B. Li Z. Lei L. Li Z. Chen L.. (2013). Induction of protective immunity against *Chlamydia muridarum* intravaginal infection with the chlamydial immunodominant antigen macrophage infectivity potentiator. Microbes Infect. 15, 329–338. 10.1016/j.micinf.2013.02.001 23416214 PMC4218745

[B17] Mishori R. Mcclaskey E. L. Winklerprins V. J. (2012). *Chlamydia trachomatis* infections: screening, diagnosis, and management. Am. Fam. Physician 86, 1127–1132. 23316985

[B18] Murthy A. K. Li W. Chaganty B. K. Kamalakaran S. Guentzel M. N. Seshu J.. (2011). Tumor necrosis factor alpha production from CD8+ T cells mediates oviduct pathological sequelae following primary genital *Chlamydia muridarum* infection. Infect. Immun. 79, 2928–2935. 10.1128/IAI.05022-11 21536799 PMC3191981

[B19] O'connell C. M. Ingalls R. R. Andrews C. W. Jr. Scurlock A. M. Darville T. (2007). Plasmid-deficient *Chlamydia muridarum* fail to induce immune pathology and protect against oviduct disease. J. Immunol. 179, 4027–4034. 10.4049/jimmunol.179.6.4027 17785841

[B20] Qu Y. Frazer L. C. O'connell C. M. Tarantal A. F. Andrews C. W. Jr. O'connor S. L.. (2015). Comparable genital tract infection, pathology, and immunity in rhesus macaques inoculated with wild-type or plasmid-deficient *Chlamydia trachomatis* serovar D. Infect. Immun. 83, 4056–4067. 10.1128/IAI.00841-15 26216426 PMC4567646

[B21] Ramsey K. H. Schripsema J. H. Smith B. J. Wang Y. Jham B. C. O'hagan K. P.. (2014). Plasmid CDS5 influences infectivity and virulence in a mouse model of *Chlamydia trachomatis* urogenital infection. Infect. Immun. 82, 3341–3349. 10.1128/IAI.01795-14 24866804 PMC4136204

[B22] Sigar I. M. Schripsema J. H. Wang Y. Clarke I. N. Cutcliffe L. T. Seth-Smith H. M.. (2014). Plasmid deficiency in urogenital isolates of *Chlamydia trachomatis* reduces infectivity and virulence in a mouse model. Pathog. Dis. 70, 61–69. 10.1111/2049-632X.12086 24022847 PMC4300952

[B23] Song L. Carlson J. H. Whitmire W. M. Kari L. Virtaneva K. Sturdevant D. E.. (2013). *Chlamydia trachomatis* plasmid-encoded Pgp4 is a transcriptional regulator of virulence-associated genes. Infect. Immun. 81, 636–644. 10.1128/IAI.01305-12 23319558 PMC3584862

[B24] Sun X. Yang Z. Zhang H. Dai J. Chen J. Tang L.. (2015). *Chlamydia muridarum* induction of glandular duct dilation in mice. Infect. Immun. 83, 2327–2337. 10.1128/IAI.00154-15 25824829 PMC4432733

[B25] Wang J. Zhang Y. Lu C. Lei L. Yu P. Zhong G. (2010). A genome-wide profiling of the humoral immune response to *Chlamydia trachomatis* infection reveals vaccine candidate antigens expressed in humans. J. Immunol. 185, 1670–1680. 10.4049/jimmunol.1001240 20581152

[B26] Zhong G. Fan P. Ji H. Dong F. Huang Y. (2001). Identification of a chlamydial protease-like activity factor responsible for the degradation of host transcription factors. J. Exp. Med. 193, 935–942. 10.1084/jem.193.8.935 11304554 PMC2193410

