# Supplementary material for: The Structural Integrity of Plasmid-Encoded Pgp3 Is Essential for Induction of Hydrosalpinx by Chlamydia muridarum
Source: Front Cell Infect Microbiol. 2019 Feb 5;9:13. doi: 10.3389/fcimb.2019.00013 (PMC6370636; doi:10.3389/fcimb.2019.00013)
Supplement: Supplementary file 1 [file Image_1.pdf]

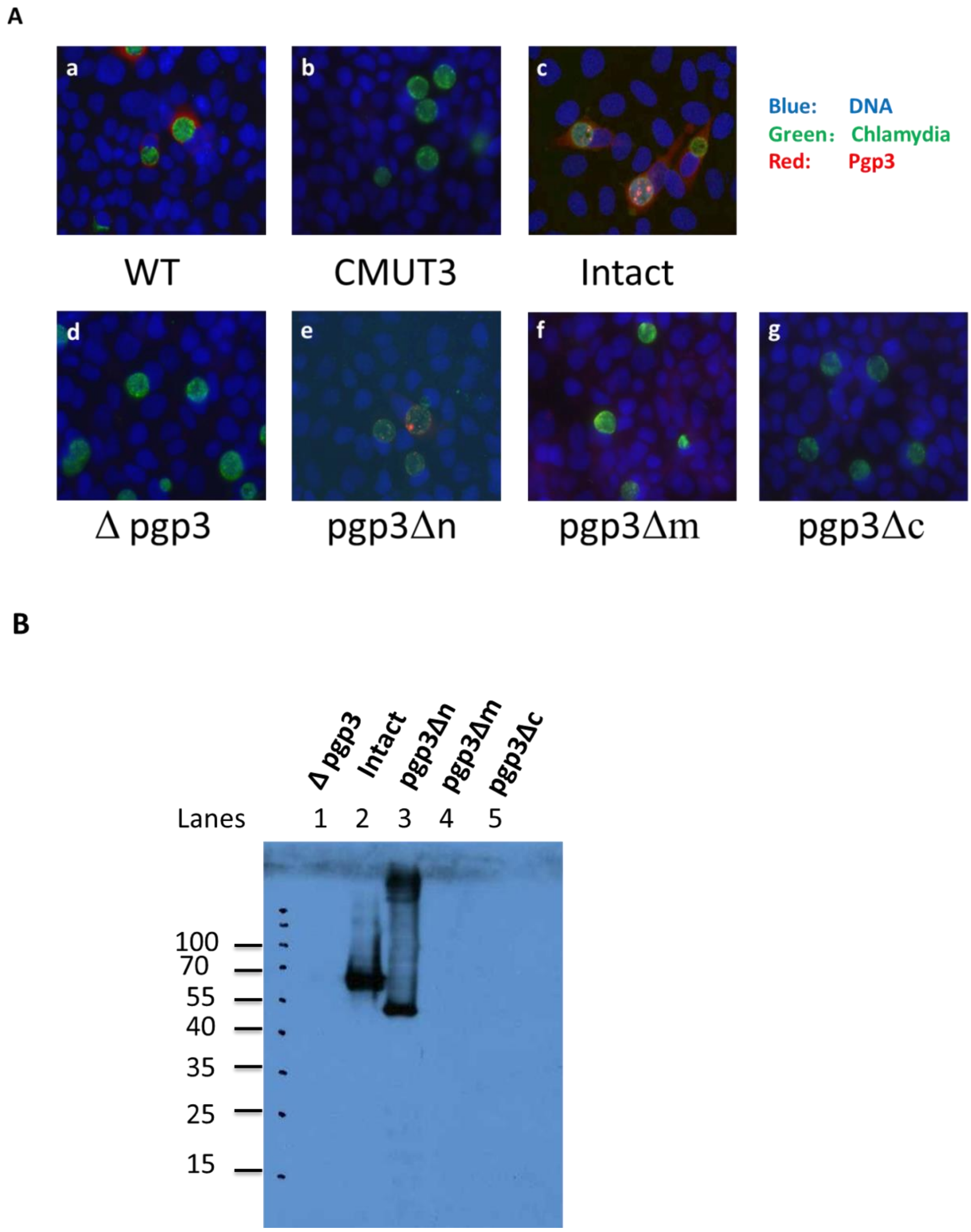

Fig. S1. Effect of Pgp3 domain deletion on polyclonal antibody recognition of Pgp3. (A). The chlamydial organisms listed below panel a-g were used to infect HeLa cells. The cells were subjected to triple immunofluorescence staining for pgp3 (red), chlamydia (green), and DNA (blue). The pictures were taken under fluorescence microscope at 40× magnification. (B). HeLa cell infected with Δ pgp3, intact organisms, or various Pgp3 mutants as indicated on top of the figure were harvested

and the cell lysates were subjected to western blotting with anti-Pgp3 polyclonal antibody under non-reducing condition. Please note that Pgp3 mutants with middle or C-terminal domain deleted failed to be recognized by Pgp3 polyclonal antibody.
