# Supplementary material for: The Structural Integrity of Plasmid-Encoded Pgp3 Is Essential for Induction of Hydrosalpinx by Chlamydia muridarum
Source: Front Cell Infect Microbiol. 2019 Feb 5;9:13. doi: 10.3389/fcimb.2019.00013 (PMC6370636; doi:10.3389/fcimb.2019.00013)
Supplement: Supplementary file 2 [file Image_2.pdf]

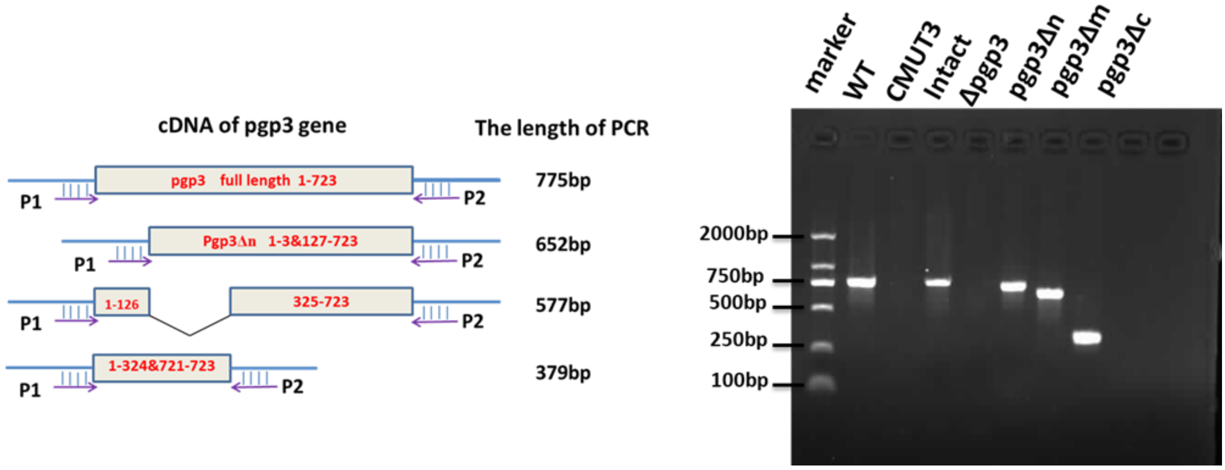

Fig. S2. **The transcription levels of different Pgp3 mutants in various transformants were assessed by RT-PCR**. HeLa cells infected with the chlamydial organisms as listed on top of the right panel were harvested at 20 h postinfection for RT-PCR detection of transcripts of the Pgp3 protein. The left panel showed the different lengths of PCR productions generated from various *C. muridarum* organisms' cDNA by one pair of primers designed from both sides of pgp3 gene. The result indicates that the mutated Pgp3 could express, at least, in the transcriptional level.
